# Prediction of the muscle strength by the muscle thickness and hardness using ultrasound muscle hardness meter

**DOI:** 10.1186/2193-1801-2-457

**Published:** 2013-09-12

**Authors:** Satoshi Muraki, Kiyotaka Fukumoto, Osamu Fukuda

**Affiliations:** Faculty of Design, Kyushu University, Fukuoka, Japan; Faculty of Engineering, Shizuoka University, Shizuoka, Japan; Measurement Solution Research Center, National Institute of Advanced Industrial Science and Technology, Saga, Japan

**Keywords:** Muscle hardness, Muscle thickness, Ultrasound, Knee extension

## Abstract

**Purposes:**

The present study investigated whether a combination of the thickness and hardness of muscles without muscle tension can be used to estimate muscle strength during knee extension in adult males and females.

**Methods:**

Seventy-two males and thirty-three females, whose ages ranged from 18 to 35 years, participated in this study. We measured muscle thickness and hardness in the right anterior region of the thigh (rectus femoris muscle and vastus intermedius muscle) without muscle tension using an ultrasound muscle hardness meter, and the maximal voluntary isometric contraction of right knee extension (MVIC). The changing ratios (%) of the tissue thickness before compression to those during compression (compression ratio) are calculated as an index of the hardness. Higher ratio indicates a harder muscle compared with that of other individuals showing the same muscle thickness.

**Results:**

In male group, although the MVIC had significantly positive correlation to both muscle thickness (r=0.412, p<0.01) and compression ratio (r=0.233, p<0.05), their variables also had correlation mutually. In the female group, the MVIC has significantly positive correlation to only compression ratio (r=0.499, p<0.01), not muscle thickness (r=0.225, n.s.). On multiple linear regression analysis, the combination of two parameters (muscle thickness and compression ratio) allowed more accurate estimation of MVIC (r=0.573, p<0.01) in the female group.

**Conclusion:**

These findings suggested that the combination of muscle thickness and hardness is capable of effectively estimating muscle strength especially in females.

## Introduction

Since the muscle strength is basis for human’s daily physical activity, its assessment is very important to let people know their level of the muscle strength especially in sedentary people. In general, however, the muscle strength test requires maximum muscle tension to tested persons (Stoll et al. 2002). In sedentary people, because their muscles and joints are not strong, muscle tension at high intensity might have possibility to injury them (Liu and Latham 2010). Accordingly, the safe testing of the muscle strength is essential especially for the sedentary people. In addition, it is preferable that the assessment can be easily conducted in the community.

On the other hand, the muscle strength is related to muscle volume which is reflected by muscle cross-sectional area and thickness (Akima et al. 2001; Freilich et al. 1995; Fukunaga et al. 2001; Maughan et al. 1983a). Because their measurements don’t need muscle tension, they are often assessed instead of the muscle strength test. However, measurement of the muscle cross-sectional area requests expensive and large-sized devices such as computed tomography (CT) and magnetic resonance imaging (MRI), and accordingly, the people have to travel to medical facilities. In contrast, the muscle thickness can be easily measured by devices of ultrasound echo. In recent devices of ultrasound echo, their technical advancement, miniaturization and price reduction will facilitate introduction of the muscle thickness measurement in the community (Fujiwara et al. 2010; Kubo et al. 2003; Wakahara et al. 2010). However, muscle thickness shows lower accuracy of the prediction for muscle strength, comparing muscle cross-sectional area, although some previous studies have been trying to predict muscle cross-sectional area from the muscle thickness (Akagi et al. 2008; Miyatani et al. 2004; Miyatani et al. 2002). In order to raise the accuracy, other indexes reflecting muscle strength should be added to that of muscle thickness.

Muscle strength depends on muscle quality as well as muscle quantity (e.g. muscle volume including muscle thickness). In other words, muscle strength is roughly determined by the product of muscle volume and muscle strength relative to muscle volume. The latter factor would reflect muscle quality, and differs among individuals in association with factors such as gender, age, and training status (Akima et al. 2001; Ogawa et al. 2012; Pinto et al. 2013). Although many previous studies pointed out the importance of muscle quality in relation to muscle strength, there have been no assessments of muscle quality without requiring muscle tension, as far as we know. If muscle quality can be assessed by some property of the muscle, it would be useful to predict muscle strength in combination with muscle thickness. Accordingly, we here focus on the possibility of using muscle hardness as an index of muscle quality.

Muscle hardness has often been measured using a tissue hardness meter. In the case of conventional tissue hardness meters, the device applies a given force on the skin, then it measures the reaction force or displacement of tissue thickness to output the tissue hardness (Horikawa 2001; Kashima et al. 2006; Murayama et al. 2000). When it is used to measure tissue hardness under the skin, it evaluates the hardness in the whole tissue between the skin surface and the bone, in other words, the combination of subcutaneous fat and muscle. Recently, Fukuda et al. (2007) developed a new small device using an ultrasound signal, which can measure the thickness and hardness in subcutaneous fat and muscle under the skin simultaneously. This device can distinguish borders between tissue layers comprising subcutaneous fat, muscle and bone using pulse echoes. The device compresses the skin surface at a constant force and emits ultrasonic waves, simultaneously. Information from immediately before the force provides values of muscle thickness. Then, information about the borders before and after compression provides values of displacement of muscle thickness, which might become an index of muscle hardness. Furthermore, this device has the advantage of measuring muscle thickness and hardness at the same time, which reduces the time and effort for measurements.

Therefore, the present study examined the relationship between muscle hardness in the anterior thigh and muscle strength of the knee extension in young adult males and females using the new ultrasound muscle hardness meter, and then investigated whether a combination of the thickness and hardness of muscles can be used to estimate the muscle strength more accurately than when using muscle thickness alone.

## Methods

### Participants

Seventy-two males and thirty-three female whose ages ranged from 18 to 35 years old, and whose body mass index ranged from 17.5 to 25, participated in this study. No participant had any orthopedic pain and injury of the lower limbs and the trunk. Written informed consent was obtained from all participants before starting the study. This study was approved by the Research Ethics Committee in Faculty of Design at Kyushu University.

### Measurements of muscle thickness and hardness

On the day of the measurements, the participants refrained the vigorous physical activity until the measurements were started. Before the measurements, the position at the middle of the thigh was detected by the position of the greater trochanter major and the lateral intercondylar tubercle, and it was marked on the skin using a pen marker. During the measurements, they kept supine position with the extension of the hips and knees on a horizontal bed whose surface has soft cushion, and were asked to take relax of the whole body. The direction of the center of the knee was adjusted to face upward using the assistant’s help or a soft small cushion.

**Figure 1:**
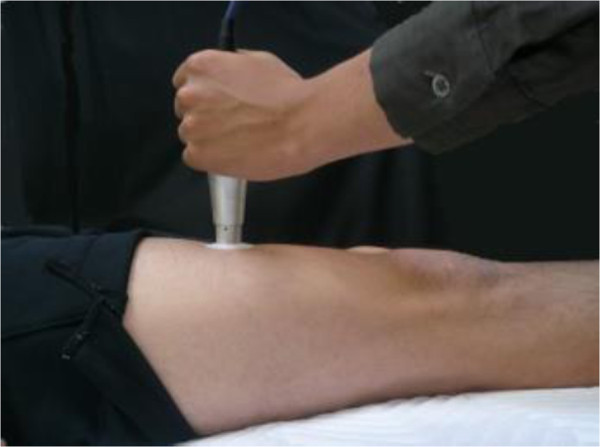
**Postures for measuring muscle hardness in the anterior thigh.**

**Figure 2:**
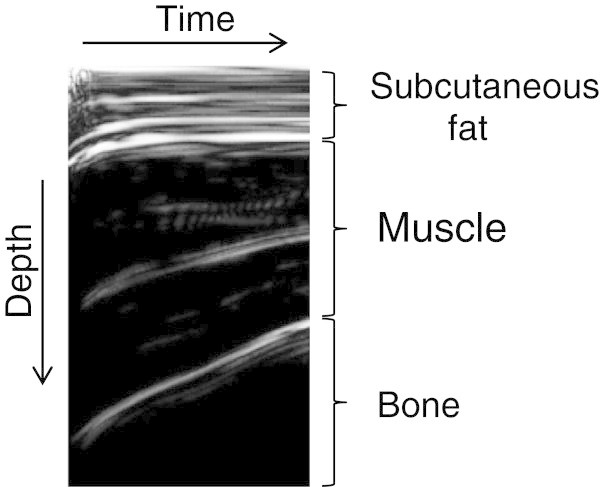
**An image obtained by the elasticity measuring instrument using an ultrasound signal.**

**Figure 3:**
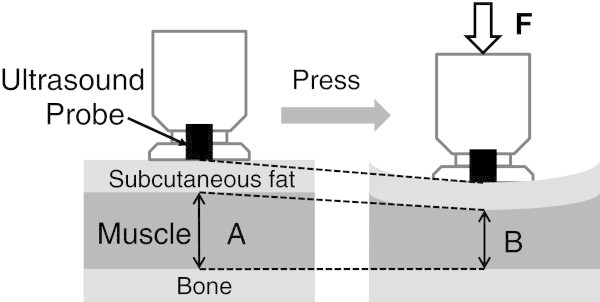
**Mechanism for measuring hardness of individual tissues by elasticity measuring instrument using an ultrasound signal.**

Tissue thickness and hardness were measured in the subcutaneous fat and the muscle group (rectus femoris muscle and vastus intermedius muscle) of the right anterior thigh using the ultrasound muscle hardness meter, an elasticity-measuring instrument that uses an ultrasound signal, as described by Tsubai et al. (2008) in detailed. In brief, the system consists of an ultrasound sensor unit, a sensor driver and a personal computer with software that controls the main unit and processes signals. An examiner presses the head of the ultrasonic sensor perpendicular to the body surface at a constant force of 10 N maintained by an internal coiled spring (Figure 1). The ultrasound muscle hardness meter is equipped with a coil spring and photo interrupter to detect the compression force limit (10 N). This mechanism ensures that the amount of force applied is constant. The thicknesses of the subcutaneous fat and the muscle group are monitored from pulse echoes reflected by tissue boundaries. Changes in thickness between before and during the compression are measured for each tissue (Figure 2). The changing ratios of the tissue thickness before compression to those during compression (compression ratio) are calculated as an index of the hardness. Higher compression ratio (nearer 100%) means higher hardness of the tissue. Figure 3 shows a model of thickness and hardness measurements of soft tissues using the non-invasive ultrasound muscle hardness meter. At each participant, mean thickness and hardness were calculated from means of three determinations at least.

### Muscle strength test

Participants underwent the muscle strength test of maximum voluntary isometric contraction by knee extension (MVIC) at a knee angle of 90 degree, as described by previous studies (Andrews et al. 1996; Demura and Demura 2013; Sipila et al. 1996). After physically warm-up, they sit at 90 degree of the hips and knees flexion on a chair without back support. A strain gauge-type tension sensor (T.K.K.1269f, Takei Scientific Instruments Company, Ltd., Nigata, Japan) was installed at the right ankle to measure the maximum force. Both their thighs were tightly strapped with a belt into the chair. After they crossed their upper arms in front of their chest, they extended the right knee with maximum effort. They were asked not to move their upper body. The test was conducted twice and the higher value is used for analysis.

### Statistical analysis

All data are presented as mean ± standard deviation (SD). The differences between male and female were tested using Student’s two-sample t-test. A simple regression analysis was performed to calculate Pearson’s product–moment correlation coefficients between the parameters analyzed. Multiple regression analysis was conducted to make formulas for predicting MVIC from parameters of muscle thickness and compression ratio. All statistical analysis was performed using SPSS for Windows version 15.0 (SPSS Inc). The statistical significance was set at p<0.05.

## Results

Physical characteristics and MVIC of the participants are presented in Table 1. Using two-sample *t-tests*, male participants were significantly taller, and had significantly greater body mass than female participants. In addition, male participants showed significantly higher absolute MVIC, its relative to body mass, and its relative to whole muscle thickness than female participants.

**Table 1:** **Physical characteristics and MVIC of participants**

	Male	Female	Significance
n	72	33	
Age (years)	23.3 ± 3.2	23.7 ± 4.7	n.s.
Height (m)	1.73 ± 0.07	1.58 ± 0.05	p<0.01
Body mass (kg)	65.6 ± 7.7	50.8 ± 4.7	p<0.01
BMI (kg·m^-2^)	21.9 ± 1.8	20.3 ± 1.5	p<0.01
MVIC (kgf)	51.8 ± 12.9	24.5 ± 7.6	p<0.01
MVIC/Body mass (kgf·kg^-1^)	0.791 ± 0.184	0.482 ± 0.140	p<0.01
MVIC/Thigh muscle thickness (kgf·mm^-1^)	1.43 ±0.35	0.81 ± 0.26	p<0.01

MVIC: Maximal voluntary isometric contraction.

Table 2 presents changes in tissue thickness by 10 N force in the anterior thigh in both sexes. In thickness before and during force, the male group indicated significantly larger thickness of the muscles and lower thickness of the subcutaneous fat than the female group, although there was no significant difference in the thickness of the whole tissue. The muscle tissue showed lower compression ratio than the subcutaneous fat, and the rectus femoris muscle showed greater lower compression ratio than the vastus intermedius muscle in both sexes. Comparing between the sexes, the male group showed significantly higher compression ratio than the female group in every tissue, except for whole tissue.

**Table 2:** **Thickness changes by 10N force in the anterior thigh**

Variables	Male	Female	Significance
Thickness without force (mm)			
Subcutaneous fat (F)	6.8 ± 1.8	11.4 ± 2.4	p<0.01
Rectus femoris muscle (R)	19.4 ± 3.2	16.6 ± 3.9	p<0.01
Vastus intermedius muscle (V)	17.2 ± 3.2	14.4 ± 3.6	p<0.01
Whole muscle (R+V)	36.6 ± 5.6	31.0 ± 6.3	p<0.01
Whole tissue (F+R+V)	43.4 ± 5.9	42.4 ± 6.8	n.s.
Thickness with 10N force (mm)			
Subcutaneous fat (F)	6.4 ± 1.6	10.5 ± 2.1	p<0.01
Rectus femoris muscle (R)	13.3 ± 3.0	10.8 ± 3.2	p<0.01
Vastus intermedius muscle (V)	14.0 ± 2.7	11.0 ± 2.4	p<0.01
Whole muscle (R+V)	27.3 ± 4.9	21.8 ± 4.5	p<0.01
Whole tissue (F+R+V)	33.7 ± 5.2	32.3 ± 5.0	n.s.
Compression ratio (%) ^1)^			
Subcutaneous fat (F)	95.5 ± 2.4	92.8 ± 3.6	p<0.01
Rectus femoris muscle (R)	67.7 ± 6.2	64.2 ± 6.1	p<0.01
Vastus intermedius muscle (V)	81.7 ± 5.0	77.3 ± 7.2	p<0.01
Whole muscle (R+V)	74.3 ± 3.6	70.4 ± 3.8	p<0.01
Whole tissue (F+R+V)	77.6 ± 3.0	76.4 ± 2.9	p<0.1

^1)^ The changing ratios of the tissue thickness before compression to those during compression.

Table 3 showed correlation coefficients between the muscle strength of the MVIC (absolute value) and each parameter regarding tissue thickness and compression ratio in the anterior thigh. In the male group, muscle thickness before (without) the compression had significant positive correlations to the strength (r=0.412, p<0.01), although the female group failed to show significant correlation to muscle thickness before the compression. In contrast, the female group showed significant correlation between the MVIC and the compression ratio of the whole muscles to force (r=0.499, p<0.01), and the coefficient was higher than that of the male group (r=0.233, p<0.05).

**Table 3:** **Correlation coefficients of thickness changes by 10N force in the anterior thigh to MVIC of knee extension**

Variables	Male	Female
Thickness without force (mm)		
Subcutaneous fat (F)	-0.018	-0.196
Rectus femoris muscle (R)	0.394**	0.237
Vastus intermedius muscle (V)	0.329**	0.135
Whole muscle (R+V)	0.412**	0.225
Whole tissue (F+R+V)	0.390**	0.139
Thickness with 10N force (mm)		
Subcutaneous fat (F)	0.003	-0.185
Rectus femoris muscle (R)	0.415**	0.293
Vastus intermedius muscle (V)	0.315**	0.285
Whole muscle (R+V)	0.420**	0.362*
Whole tissue (F+R+V)	0.399**	0.251
Change ratio to 10N force (%) ^1)^		
Subcutaneous fat (F)	0.209	0.139
Rectus femoris muscle (R)	0.303**	0.275
Vastus intermedius muscle (V)	-0.053	0.314
Whole muscle (R+V)	0.233*	0.499**
Whole tissue (F+R+V)	0.211	0.400*

^1)^ Percentage of thickness during force to that before force. *p<0.05, **p<0.01.

**Figure 4:**
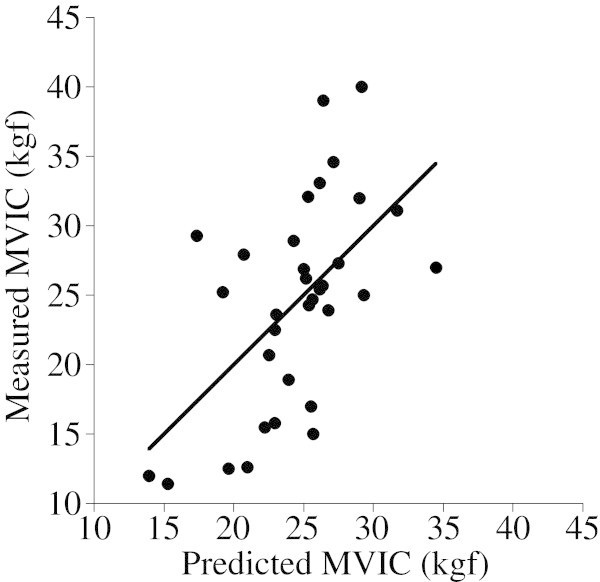
**The relationship between the predicted MVIC from the muscle thickness and compression ratio and measured MVIC in female group (n=33).**

Before conducting a multiple regression analysis for predicting the MVIC from two independent variables (thickness and compression ratio of the muscle tissue), we checked the relationship between their independent variables. The male group showed significant correlation between them (r=0.441, p<0.01), although no significant correlation was found in the female group (r=0.110, n.s.). Accordingly, we calculated a regression formula for only females as follows, and Figure 4 shows the relationship between the predicted and measured MVIC in the female group.

## Discussion

So far, some previous studies have reported measurements of muscle hardness (Horikawa et al. 1993; Kashima et al. 2004). Since it doesn’t request muscle tension and can measure at relax posture, participants are out of risk, pain and fatigue while measurement. Furthermore, most of devices for muscle hardness are small and light, which make possible to carry around. In addition, measurement by the ultrasound muscle hardness meter can simultaneously measure both muscle thickness and hardness, which save the measurement time and labor. The measurement of muscle hardness would have a potential to spread as tool for assessment of muscle ability in the field of health promotion.

In the anterior thigh, the subcutaneous fat and two muscles (rectus femoris muscle and vastus intermedius muscle) are located on the thigh bone. In both sex groups, the compression ratios are larger in the muscle tissues than the subcutaneous fat tissue, despite that the muscle is the inner side of the subcutaneous fat. When the tissue is pressed by a given force from the surface, the force is attenuated in deeper layer. Consequently, the compression ratio becomes smaller in the inner layer than outer layer if the hardness is uniform throughout the tissue. Based on these laws, lower compression ratio of the muscle means that the muscle is less hard (in other word, softer) than the subcutaneous fat. On the other hand, the vastus intermedius muscle indicated higher compression ratio than the rectus femoris. Because the vastus intermedius muscle is located at the inner side of the rectus femoris muscle. Therefore, the higher compression ratio in the vastus intermedius muscle would be mainly due to attenuation of force. Moreover, this theory can also explain the significantly higher compression ratio in the male muscles because the higher muscle thickness would attenuate the force applied by the muscle hardness meter.

In the male groups, the muscle thickness was significantly correlated to the MVIC. Many previous studies have been reporting the positive relationships of muscle cross-sectional area and thickness in the thigh to muscle strength and torque of knee extension in male adults. However, their correlation coefficient ranged between around 0.5 to 0.8 (Akima et al. 2001; Freilich et al. 1995; Kanehisa et al. 1994; Maughan et al. 1983b). The correlation coefficient in the present study was 0.412, which was relatively lower than those of other studies. Freilich et al. (1995) reported correlation coefficient (r) of 0.55 (n=58). The range of the thickness in their participants (about 25 mm to 65 mm) is larger than those in the present study (23.6 to 47.5 mm). The correlation coefficient would increase if the wider distribution in the muscle thickness is adopted.

In the female group, no significant correlation was found between muscle thickness and MVIC. Although many previous studies have been reporting significant correlation coefficient between muscle cross-sectional area in the thigh and maximal muscle strength or peak torque of knee extension in sex groups, the coefficients were lower in female group than male group (e.g. Akima et al. (2001): male r=0.827, female r=0.657, Maughan et al. (1983b): r=0.59, female r=0.51, Kanehisa et al. (1994): male r=0.707, female r=0.636). Furthermore, the maximal muscle tension against a give muscle area is pointed out to be lower in female, comparing male (Akima et al. 2001; Kanehisa et al. 1994). The present study also showed same trends that muscle strength against a given muscle thickness was significantly lower in female. This sex difference is considered to be oriented by lower contribution of muscle quantity to muscle strength in female, as described in next paragraph.

Some previous studies reported that female adults showed lower increase in muscle mass (Walts et al. 2008) and muscle fiber cross-sectional area (Martel et al. 2006) upon muscle strength training, compared with male adults. Delmonico et al. (2005) suggested that strength training-induced increase in peak power depends on muscular hypertrophy in men, but not in women. In addition, Yasuda et al. (2005) using short-term limb immobilization found that loss of isometric knee extensor peak torque was highly correlated with atrophy in the cross-sectional area of the entire quadriceps muscles for males, but not for females. On the basis of these previous findings, the change of muscle strength in females would not be more strongly accompanied by a change of muscle quantity including muscle thickness, compared with that in males. This suggests that muscle quality in females would contribute more to muscle strength, which was supported by the results of a higher correlation between compression ratio and muscle strength.

The female group showed a higher correlation coefficient between muscle compression ratio and MVIC, compared with the male group. Muscle strength is determined by not only muscle quantity, such as measured by muscle thickness in the present study, but also muscle quality. In females, muscle compression ratio was found to be independent of muscle thickness, and had a relationship to muscle strength. Namely, it is highly possible that a muscle’s compression ratio reflects muscle quality. In the present study, because the muscle hardness was measured in a supine position without knee extension and muscle tension, the anterior thigh muscle was not stretched. Accordingly, the compression ratio would reflect the hardness itself of the muscle tissue against the direction of compression by the ultrasound hardness meter. It is considered that females with higher muscle strength had superior qualitative properties in the muscles, instead of muscle hypertrophy. Some previous studies reported that isometric strength and isokinetic peak torque of knee extension are associated with ultrasound echo intensity and suggested that these relationships are caused by the difference in the amount of connective and adipose tissues in the muscles (Cadore et al. 2012; Fukumoto et al. 2012). Thus, the tissue composition of muscle might differ in association with the levels of muscle strength, which reflect the muscle hardness against a force perpendicular to the direction of the muscle fibers, although the mechanism involved is not clear from these findings.

In females, the combination of muscle thickness and muscle compression ratio increased the predictability of muscle strength from r=0.225 (only muscle thickness) to r=0.573. Although this predictability is not necessarily high, it corresponds to that by muscle cross-sectional area (e.g. Maughan et al. (1983b): female r=0.51, Kanehisa et al. (1994): female r=0.636). Considering the convenience of the measurements, the assessment of these combinations using the ultrasound muscle hardness meter would be more useful for adults to promote their health in the community, compared with that of muscle cross-sectional area by MRI and CT. Assessment using the ultrasound muscle hardness meter should also become an effective method for the middle-aged and elderly who are subjected to a greater risk in muscle strength tests. Future research should be directed toward other female age groups. In contrast, in males, the above combination was not effective to estimate muscle strength because the two variables were linked to each other. As previously described, the force applied by a hardness meter is attenuated in deeper layers. Because the male group had greater muscle thickness, their compression ratio was significantly higher than in the female group. Accordingly, the compression ratio in males might more strongly reflect the influence of muscle thickness, for example, force attenuation, not muscle hardness itself. The present study used a force of 10 N to compress the tissue. This force might be insufficient to compress the whole muscle in the male group, and might need to be adjusted for each gender.

In conclusion, these findings suggested that the combination of muscle thickness and hardness as assessed by the ultrasound muscle hardness meter is capable of effectively estimating muscle strength of knee extension without muscle contraction especially in females.
